# Wellbeing Training Based on Contemplative Practices in a Sample of Intensive Care and Homecare Professionals: A Pilot and Feasibility Non-Randomized Clinical Trial

**DOI:** 10.3390/ijerph192013137

**Published:** 2022-10-12

**Authors:** Ausiàs Cebolla, Laura Galiana, Jaime Navarrete, David Alvear, Elena Garrote, Noemí Sansó, José V. Carmona, Mar Juan, María L. Blasco

**Affiliations:** 1 Department of Personality, Evaluation, and Psychological Treatments, University of Valencia, 46010 Valencia, Spain; 2 CIBER of Physiopathology of Obesity and Nutrition (CIBEROBN), 28029 Madrid, Spain; 3 Department of Methodology for the Behavioral Sciences, University of Valencia, 46010 Valencia, Spain; 4 Institut de Recerca Sant Joan de Déu, 08950 Esplugues de Llobregat, Spain; 5 Teaching, Research & Innovation Unit, Parc Sanitari Sant Joan de Déu, 08830 Sant Boi de Llobregat, Spain; 6 Department of Developmental and Educational Psychology, University of the Basque Country-UPV/EHU, 48940 San Sebastian, Spain; 7 Department of Nursing and Physiotherapy, University of the Balearic Islands, 07122 Palma, Spain; 8 Balearic Islands Health Research Institute (IDISBA), 07120 Palma, Spain; 9 Faculty of Health Sciences, Universidad Europea de Valencia, 46010 Valencia, Spain; 10 Hospital Clínico Universitario de Valencia, 46010 Valencia, Spain

**Keywords:** contemplative positive psychology, meditation, compassion, intensive care professionals

## Abstract

Background: Intensive care unit (ICU) and homecare unit professionals are susceptible to higher levels of stress and burnout than other healthcare professionals, which has an impact on their well-being, and in turn on their patients. In terms of data, there is not much research about the effects of psychological interventions on ICU and homecare professionals. The aim of this study was to investigate the effectiveness of Wellbeing Training based on Contemplative Practices (WTCP) for the increase of psychological functioning in a sample of ICU and homecare professionals. Methods: A pilot and feasibility non-randomized clinical trial was conducted. Participants in the WTCP group (*n* = 19) attended an at-work 8-session/2 h group WTCP program aimed at directly training four basic skills: (a) sustained positive emotions, (b) recovery from negative emotions, (c) pro-social behavior and generosity, and (d) mind wandering, mindfulness, and “affective stickiness”. Nineteen professionals were allocated in the control group. Results: Results indicated that WTCP had a positive impact on self-compassion, personal accomplishment (burnout), and frequency of negative emotions. Moreover, a thematic analysis of participant interviews (*n* = 14) was conducted. Conclusions: These preliminary results are promising, though future research is needed to evaluate the effectiveness of WTCP using randomized controlled trial methodologies.

## 1. Introduction

Healthcare (HC) professionals show high levels of psychological distress and burnout [1], which impacts their well-being [2], and also has an impact on their patients and the health care system [3]. In fact, stress and burnout have been linked to increased medical errors, impact of professional qualities (e.g., lower empathy), and poorer patient satisfaction [4]. Intensive care unit (ICU) and homecare professionals are especially susceptible to even higher levels of stress and burnout due to the stressful environment. Issues such as diagnosis, end-of-life processes, ethical decision-making, working with patients and relatives who express continuous suffering, or miscommunication conflicts have been identified as predictors of stress and burnout [5,6].

Different intervention strategies to reduce empathetic distress and burnout among HC professionals have recently been developed. On one side, authors identify work organizational interventions, and on the other, individual psychological interventions that include psychotherapy, communication skills training, educational programs, and meditation or contemplative practice-based interventions, including mindfulness-based interventions (MBIs) and compassion-based interventions (CBIs). In a recent systematic review [7], both MBIs and CBIs showed interesting results in HC workers. For example, MBIs were effective in terms of stress and safety compliance at work [8]. However, the efficacy of MBIs in decreasing burnout in HC professionals has been recently disputed in a systematic review, showing that they are not so effective as expected [9]. Indeed, recent studies in HC workers have shown no effect on stress or that the effect was not maintained over time [10].

Despite both MBIs and CBIs appearing to decrease these specific negative aspects, they have also shown to be effective at increasing positive aspects such as well-being [11], quality of life [12], or positive emotions [13]. However, this aspect has received less attention compared to symptom reduction. Regarding the ICU, there is not much research about the effects of MBIs. Only a few studies have shown promising results [14,15], and no previous study has been developed to test the efficacy of other contemplative practices beyond MBIs. Thus, there is a need to increase the research and programs adapted to this population.

In searching for new perspectives, researchers suggest the need to generate a dialogue between contemplative practice-based interventions and other proximal areas, such as positive psychology (PP) [16,17]. To embrace both paradigms, the concept of contemplative positive psychology has been developed, understood as “the area of PP that includes a range of techniques and conceptualizations developed by the contemplative sciences for the promotion of well-being through evidence-based strategies” [18]. From this perspective, a novel program called Wellbeing Training based on Contemplative Practices (WTCP) [19] has been developed. 

WTCP was inspired by the Self-Centeredness/Selflessness Happiness Model [20] in which authentic happiness is related to selflessness psychological functioning, also known as hypoegoic functioning [21]. WTCP proposes increasing this psychological functioning through the training of four basic skills, identified by Davidson and Schuyler [22] as the constituents of happiness: (a) sustained positive emotions, (b) recovery from negative emotions, (c) pro-social behavior and generosity, and (d) mind wandering, mindfulness, and “affective stickiness”. The program, compared to traditional MBIs, focuses on different meditation family strategies, mainly generative or constructive ones. Furthermore, it puts special emphasis on virtuous actions, emotion regulation (both positive and negative emotions) and compassion, and includes strategies from PP, such as strengths-based interventions, savoring, kindness, or the three good things. 

Considering previous research, the aim of this study was to explore the potential outcome and acceptability of a contemplative positive psychology intervention (WTCP) to increase positive emotions and self-compassion (main outcomes), and decrease burnout and difficulties in emotion regulation (secondary outcomes) in a sample of ICU and homecare professionals. 

The working hypotheses were as follows: (1) Participants of the WTCP intervention group will experiment an increase in positive emotions, and a decrease in negative emotions and difficulties in emotion regulation, when compared to the control group, and (2) as emotion regulation has been related to self-compassion and burnout, participants of the WTCP intervention group will experience an increase in the positive facets of self-compassion and burnout (i.e., positive self-compassion and personal acceptance), and a decrease in the negative dimensions of these variables (i.e., negative self-compassion, depersonalization, and emotional exhaustion), when compared to the control group.

## 2. Materials and Methods

### 2.1. Trial Design

A pilot and feasibility non-randomized clinical trial was conducted. We followed the CONSORT [23] guidelines for reporting non-randomized pilot and feasibility studies [24]. 

### 2.2. Participants

The participants were recruited throughout different presentations in the ICU and homecare units of two hospitals in Valencia (Spain), where the main topics of the intervention were explained. The control group was recruited through announcements in the same hospitals. Participation was voluntary, and after signing their consent, participants were invited to answer the battery of questionnaires. Randomization was not possible due to the characteristics of the ICU and homecare units (shifts, space for completing the training, etc.). Once the intervention was completed, subjects of the intervention group were invited to participate in individual semi-structured interviews conducted by an independent researcher. After participants provided verbal consent, the independent researcher recorded the interviewees’ answers.

Qualitative data collection was the following procedure. After the end of the intervention, participants of the intervention group were invited to participate in an individual semi-structured interview. Participants gave their verbal consent, after which the interview was conducted. The interview consisted of six open-ended questions, three of which are analyzed in this study. These questions fall under the research question “What benefits has the participant gained from the WTCP intervention?”. The interview was recorded under private conditions, with the consent of the participant, and ended spontaneously when they said they had nothing more to say.

In regard to the eligibility criteria for participants, inclusion criteria were as follows: (a) over 18 years of age; (b) healthcare professional working in intensive care units or homecare units; and (c) have signed the informed consent document and confidentiality agreement.

### 2.3. Intervention

WTCP was composed of 8 sessions in a 2 h session format through psychoeducation, group dynamics, narrative exercises and guided meditations. Participants received an activity book where they could review the theoretical aspects and the tasks for each session. In between class sessions, participants were encouraged to complete the home activities and tasks and meditate daily using the meditations uploaded to a web (programaebc.com). The training protocol was sequential and iterative. The sessions were: (1) introduction to the program: motivations and intentions to train well-being, (2) mindfulness, (3) savoring, (4) signature strengths, (5) regulation of negative emotions, (6) compassion and altruism, (7) multidimensional self and finally, the last session (8), where participants talked about the future and the implementation of the changes proposed by the program. The intervention was delivered on site in the ICU, during working hours. The WTCP instructors were a clinically trained psychologist, researchers, and experienced meditators. The sessions were supervised by the WTCP training supervisor team. A table that summarizes the schedule of the agenda and topics covered is included as Supplementary Material (Table S1).

### 2.4. Outcomes

Measures were obtained on all study participants (WTCP and control) at two time points: recruitment (pre-test) and two months after baseline evaluation (post-test). Data were collected on satisfaction, acceptance, and adherence to the WTCP program and demographic factors.

For the main outcomes, positive emotions and self-compassion, we used the following measurement instruments:

- Modified Differential Emotions Scale (mDES) [25]. This questionnaire measures 20 different emotions in a period of 15 days. Ratings were made on a 5-point scale (0 = never, 4 = most of the time). The Positive Emotions subscale is a composite of 9 positive emotions with coefficient α = 0.79. The Negative Emotions subscale is a composite of 7 negative emotions with coefficient α = 0.69.
- Self-Compassion Scale—Short Form (SCS-SF) [26]. This is a 12-item questionnaire designed to assess overall self-compassion (total score) and three self-compassion facets, developed to measure how patients relate to themselves when things go wrong. Ratings were made on a 5-point scale, from 1 (almost never) to 5 (almost always). Although the original structure establishes that there are three different factors [27], some authors propose that positive (positive self-compassion) and negative items (negative self-compassion) work better as a factor underlying the SCS [28]. The Spanish version of the SCS-SF has shown high internal consistency and high test–retest reliability [29]. In this study, reliability estimates were α = 0.78 for positive self-compassion and α = 0.86 for negative self-compassion.

For the secondary outcomes, burnout and difficulties in emotion regulation, the following measurement instruments were used:

- Difficulties in Emotion Regulation Scale-18 (DERS-18) [30]. This is a measure of emotion (dys-)regulation ability developed by Gratz and Roemer [31] but in its short form. Rated on a scale from 1 (almost never) to 5 (almost always), the scale is composed of five factors: Goals, Non-acceptance, Impulse, Clarity, Awareness, and Limited Access to Emotion Regulation Strategies. The DERS-18 exhibited high internal consistency, as well as strong convergent and concurrent validity by showing relationships. In this study, reliability estimates were α = 0.81 for Goals, α = 0.76 for Non-acceptance, α = 0.86 for Impulse, α = 0.91 for Clarity, α = 0.73 for Awareness, and α = 0.84 for Limited Access to Emotion Regulation Strategies.
- Maslach Burnout Inventory (MBI) [32]. This questionnaire measures the level of perceived burnout through twenty-two items with a seven-point Likert-scale, ranging from 0 (never) to 6 (every day). The instrument consists of three subscales: Personal Accomplishment, Emotional Exhaustion, and Depersonalization. The Spanish version, which showed high internal consistency, was used [33]. In this study, reliability estimates were α = 0.73 for Personal Accomplishment, α = 0.87 for Emotional Exhaustion, and α = 0.71 for Depersonalization.

In order to measure usefulness and acceptability, a set of statements was developed with a score range from 1 (do not agree at all) to 5 (agree). To measure usefulness, the indicators used were: “This training will help me manage my job better”, “I have learned tools and resources to improve my attention and concentration”, “I have learned skills to manage my emotions healthy”, and “I have learned valuable tools and resources to manage stress”. To measure acceptability, the following indicators were used: “I would recommend this course to other people with the same profession” and “I would recommend this course to everyone”.

### 2.5. Sample Size

After reviewing prior research on the effectiveness of interventions in MBIs for reducing stress in healthcare professionals, we found the result of the meta-analysis carried out by Burton et al. [34]. According to these authors, the combined effect size was *r* = 0.342. Taking this as the effect size (converted to *F*), a sample size of 38, with 19 in each group, was selected so that we could maintain a 10% Type II error rate and a 5% Type I error rate in mixed multivariate analyses of variance (MANOVA), with 2 groups, and with the aforementioned effect size.

### 2.6. Statistical Methods

The data were double entered and checked for accuracy. All of the values for univariate skewness and kurtosis for all the variables analyzed were satisfactorily within conventional criteria for normality. Given that missing values were less than 5%, they were not considered to cause bias in estimates [35]. Therefore, no adjustments were made to the scores for the variables measured in our study.

First, groups were compared according to their sociodemographic characteristics and main outcome variables at baseline level. The Chi-squared test and t-tests for independent samples were used for this purpose. Second, and in order to study the effectiveness of the intervention on difficulties in emotion regulation and positive and negative emotions (Hypothesis 1), two mixed multivariate analysis of variance (MANOVA) were carried out. In both of them, the within-subjects independent variable was time, with two categories (pre- and post-intervention), and the between-subjects independent variable was the group, also with two categories (control vs. intervention group). The first MANOVA included the dimensions of DERS-18 as dependent variables. In the second MANOVA, the dependent variables were the dimensions of m-DES: positive and negative emotions. 

In order to check Hypothesis 2, two mixed-multivariate analysis of covariance (MANCOVA) were calculated. In both of them, the within-subjects independent variable was time, the between-subjects independent variable was the group, also with two categories (control vs. intervention group). In the first MANCOVA, dependent variables included the dimensions of burnout: depersonalization, emotional exhaustion, and personal acceptance. In the second, dependent variables were the dimensions of self-compassion: positive and negative self-compassion. In regard to the covariates, gains in difficulties in emotion regulation, positive emotions, and negative emotions between the pre-post intervention time points for both groups were included. That is, the main effects and interaction of the independent variables (time and group) were assessed after scores in burnout and self-compassion were adjusted for differences associated with the covariates, as changes in burnout and self-compassion were theoretically attributed to the improvement in emotion regulation. 

In the MANOVAs and MANCOVAs, the effect of time indicates if there is a change, whether positive or negative, over time; the effect of group indicates if there are differences in the general mean between the groups, without taking into account the temporal point; the effects of the covariates, when included, indicate a statistically significant relationship between the dependent variables and the covariates: and the effect of the interaction time*group will be the one that indicates if there are any differences due to the intervention. Taking into account the proposed hypotheses, the effect of the interaction will respond to Hypotheses 1 and 2. Within the different multivariate criteria to study the effects of the independent variables, we chose Pillai’s criteria, the most robust criteria for violations of statistical assumptions [35]. The effect size was estimated with partial eta-squared (*η*^2^). Cohen’s effect size cut-offs criteria were used for descriptive ends: 0.02, 0.13, and 0.26, for small, medium, and large effects, respectively [36].

When analyzing the responses obtained in the semi-structured interview, we opted for a qualitative analysis with a particularly inductive character, searching for underlying categories from the data obtained. This is a bottom-up approach to data analysis, in which the authors primarily use the empirical value of the data obtained from the participants’ responses. For this purpose, we followed the method used in the thematic analysis [37] in order to analyze the responses to the open-ended question. This method allows us to identify, organize, analyze in detail, and provide patterns or themes from a careful reading and re-reading of the collected information, and thus infer results that are conducive to the proper understanding/interpretation of the phenomenon under study. Data analysis was carried out by two team members: psychologists with expertise in contemplative sciences and/or qualitative methods. The six phases of analysis we followed were [37]: (1) familiarization with the data; (2) generating initial codes, each code involving a brief labelling that captures the essence of the meaning unit; (3) searching for themes; (4) reviewing themes, each theme being reviewed and organized in a coherent pattern (a coherent pattern includes internal homogeneity (i.e., codes are conceptually integrated in a meaningful way within each theme) and external homogeneity; subsequently, the team re-examined all definitions as a whole to ensure that all relevant meaning units were captured by one of the themes); (5) defining and naming themes; and (6) creating the report.

Reliability was obtained by inter-coder agreement, the product of systematic reflections to define and establish the codes (sub-codes) and categories (sub-categories). Validity was obtained during this inter-coder agreement, in which a coder, external to the research project, agreed to assign the same analysis codes in the same categories coded by the research team [38].

## 3. Results

### 3.1. Participant Flow

**Figure 1:**
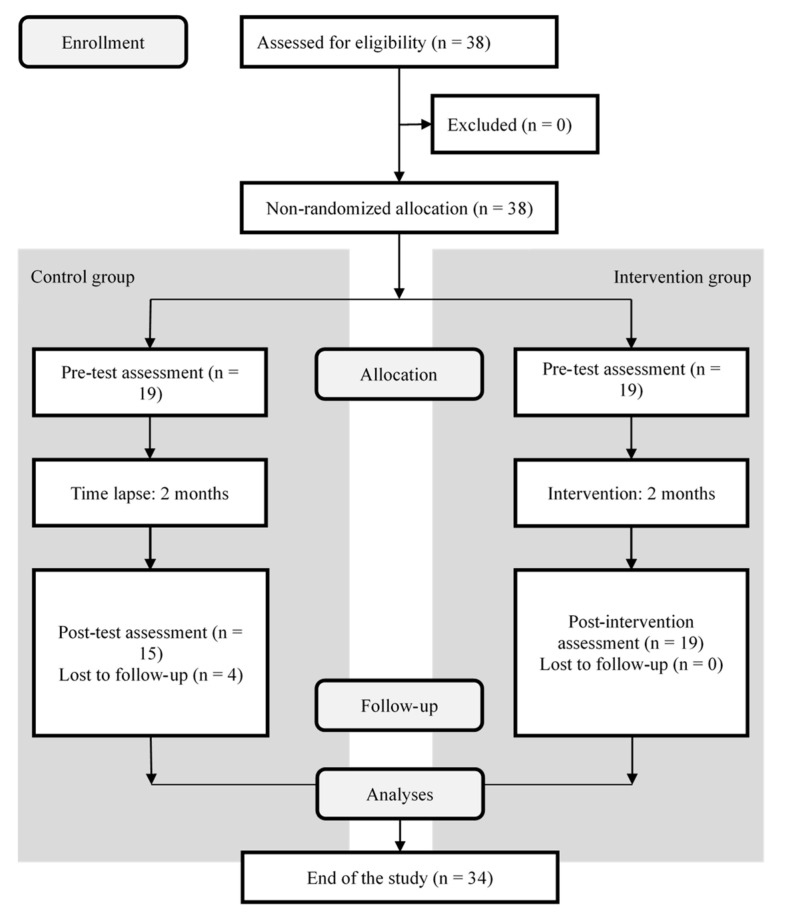
CONSORT flow diagram of the study.

A total of 19 healthcare professionals applied to participate and attended the first session; all of them completed the training course and the pre- and post-test. Regarding the control group, 19 healthcare professionals consented to being part of the study, but only 15 completed the pre- and post-test. Of the 19 subjects who completed the course, 17 attended six to eight sessions and two missed four sessions. A flow diagram showing the participants’ progression through the study is shown in Figure 1.

### 3.2. Baseline Data

Table 1 shows the sociodemographic characteristics of both the intervention and the control group.

Groups were compared according to their sociodemographic characteristics and main outcome variables at baseline level. Groups showed no differences regarding gender distribution (*Fisher’s exact test p =* 1.000), nor differences in age (*t*(32) = 0.864, *p* = 0.394). Regarding the comparison of the main outcome variables, no statistically significant differences were found, except for the levels of strategies (DERS-18), which were higher for the control group, and positive emotions (m-DES) and personal accomplishment (MBI), which were higher for the intervention group (see Table 2). However, in the analyses testing the intervention effect, the influence of differences in the pre-intervention time point were controlled for.

Regarding adherence, participants attended an average of 6.4 sessions (SD = 1.67), they practiced meditation at home an average of 3.8 (SD = 1.6) days per week, and the average minutes per practice session was 23.1 (SD = 14.5).

### 3.3. Hypotheses Testing

The first MANOVA studied the effect on the dimensions of the DERS-18. Results showed a statistically non-significant effect size for time (Pillai’s trace= 0.321; *F*(6,27) = 2.130; *p* = 0.083; *η*^2^ = 0.321), group (Pillai’s trace = 0.333; *F*(6,27) = 2.244; *p* = 0.069; *η*^2^ = 0.333), the interaction time*group (Pillai’s trace = 0.157; *F*(6,27) = 0.836; *p* = 0.553; *η*^2^ = 0.157). Therefore, the results revealed no effectiveness of the intervention at improving difficulties in emotion regulation. As shown in Table 3, follow-up ANOVAs showed no effects of the training on the DERS-18 dimensions (see Table 3). 

The MANOVA studying the effect of the independent variables on the dimensions of m-DES showed a statistically significant and large effect for time (Pillai’s trace = 0.688; *F*(2,30) = 33.079; *p* < 0.001; *η*^2^ = 0.688), for group (Pillai’s trace = 0.221; *F*(2,30) = 4.259; *p* = 0.024; *η*^2^ = 0.221), and for the interaction time*group (Pillai’s trace = 0.374; *F*(2,30) = 8.971; *p* = 0.001; *η*^2^ = 0.374). Therefore, the intervention improved healthcare professionals’ emotions. Specifically, and as pointed out in the follow-up ANOVAs (Table 3), the intervention succeeded at decreasing the negative emotions of healthcare professionals, who showed a higher decrease in this dimension when compared to the professionals of the control group (see Table 4). 

The MANCOVA studying the effect of the independent variables on the dimensions of burnout showed a non-statistically significant effect for time (Pillai’s trace = 0.093; *F*(3,26) = 0.886; *p* = 0.461; *η*^2^ = 0.093) and the interaction time*group (Pillai’s trace = 0.143; *F*(3,26) = 1.449; *p* = 0.251; *η*^2^ = 0.143), but a statistically significant effect for group (Pillai’s trace = 0.549; *F*(3,26) = 10.571; *p* < 0.001; *η*^2^ = 0.549). The follow-up ANOVAs, however, showed a statistically significant effect of the interaction time*group on the personal accomplishment dimension (see Table 3). Specifically, a decrease in personal accomplishment was observed in both the control and the intervention group, but this decrease was higher for those who were not enrolled in the intervention (see Table 4). Regarding the covariates, a statistically significant effect was found for emotional regulation gain (Pillai’s trace = 0.320; *F*(3,26) = 4.079; *p* = 0.017; *η*^2^ = 0.320) and negative emotions (Pillai’s trace = 0.405; *F*(3,26) = 5.906; *p* = 0.003; *η*^2^ = 0.405), but not for positive emotions (Pillai’s trace = 0.082; *F*(3,26) = 0.773; *p* = 0.520; *η*^2^ = 0.082). This means that the effect of the intervention on burnout was influenced by both the gain in emotional regulation and the decrease in negative emotions produced by the intervention.

The last MANCOVA studied the effect of the intervention on self-compassion and self-criticism. Results revealed non-statistically significant effects for time (Pillai’s trace = 0.041; *F*(2,27) = 0.582; *p* = 0.566; *η*^2^ = 0.041) and the interaction time*group (Pillai’s trace = 0.131; *F*(2,27) = 2.040; *p* = 0.150; *η*^2^ = 0.131), but a statistically significant effect for group (Pillai’s trace = 0.259; *F*(2,27) = 4.716; *p* = 0.025; *η*^2^ = 0.240). The follow-up ANOVAs, however, did show a statistically significant effect of the interaction time*group on self-compassion (see Table 3). In fact, whereas a slight decrease was found in the self-compassion levels of the control group, an increase of this dimension was found in the intervention group (see Table 4). Regarding the covariates, a statistically significant effect was found for negative emotions (Pillai’s trace = 0.240; *F*(2,27) = 4.264; *p* = 0.025; *η*^2^ = 0.240), but not for positive emotions (Pillai’s trace = 0.018; *F*(2,27) = 0.244; *p* = 0.785; *η*^2^ = 0.018), nor for emotional regulation gain (Pillai’s trace = 0.175; *F*(2,27) = 2.861; *p* = 0.075; *η*^2^ = 0.175). This means that intervention gains in negative emotions were related to the results in self-compassion.

### 3.4. Acceptability of the Intervention

At the end of the intervention, participants answered a survey about the acceptability and usefulness of the program. Overall, participants showed high acceptance of the intervention, and recognized that it would help them manage their job better (M = 4.5, SD = 0.7). More specifically, they reported that they had learned resources to improve attention regulation (M = 4.7, SD = 0.45), skills to a healthy management of emotions (M = 4.7, SD = 0.45), and tools to manage stress (M = 4.5, SD = 0.69). Moreover, they would recommend it not only to other professionals in similar situations (M = 4.7, SD = 0.41), but also to everyone (M = 4.3, SD = 1). 

### 3.5. Qualitative Analyses

We conducted a thematic analysis of participants’ interviews. All the participants of the intervention group were invited to participate, but only 14 agreed to participate. The rest did not attend due to lack of time. The three open-ended questions (“What benefits are obtained from the WTCP program?”, “What activities or contemplative practices have been most beneficial?”, and “How has the WTPC program helped you on a personal or professional level?”) fell under the research question “What benefits has the participant gained from the WTCP intervention?”. Six themes were identified (see Table 5 and the qualitative analysis report in the Supplementary Material, Table S1): improved emotion regulation, increased awareness, increased well-being, increased savoring, improved self-care and care for others, and knowledge acquisition.

## 4. Discussion

The aim of this study was to run a mixed-methods feasibility non-randomized pilot study to explore the potential outcome and acceptability of an 8-week novel intervention that combines contemplative and PP practices called Wellbeing Training based on Contemplative Practices (WTCP) [18] in a sample of ICU and homecare professionals compared to a control condition. Regarding Hypothesis 1, we expected that participants of the WTCP intervention group would experiment an increase in positive emotions, and a decrease in negative emotions, and difficulties in emotion regulation. Results indicate a significant decrease in the frequency of negative emotions, but there were no differences in the frequency of positive emotions or in emotion regulation. Traditional MBIs do not intentionally generate positive thoughts and feelings, however an orientation of acceptance toward present-moment experiences have an impact on positive emotions [39]. Given that WTCP is rooted in PP and has a specific model of savoring and sustaining positive emotions, we expected a change in the frequency of positive emotions. A systematic review found that compassion mediations had medium and small effect sizes on positive emotions [40]; however, none of these studies were carried out in a sample of ICU or homecare professionals, with high levels of stress, which could explain these unexpected results. Regarding difficulties in emotion regulation, previous studies have shown how emotion regulation is one of the mechanisms involved in the efficacy of MBIs [41], and in fact, mediates the relationship between self-compassion and mental health [42]. Given that burnout is characterized by emotional exhaustion, emotion regulation has been proposed as an important target, and its dysregulation a predictor of it, in HC professionals. 

Our second hypothesis expected an increase in self-compassion and burnout. The results partially supported this hypothesis; there was a significant increase in self-compassion (but not a reduction in self-criticism). Self-compassion has been found to be one of the main mechanisms of both MBIs [43] and CBIs [44]. This result is in line with the previous literature, which has found that MBIs are effective at increasing self-compassion in HC professionals [9].

Regarding burnout, the only factor which showed a significant increase was personal accomplishment, which refers to feelings of competence and successful achievement. These results are especially important, given that they refer to organization climate and could be related to the altruism and compassion modules, in which many exercises were focused on achieving altruistic and kind behaviors. The efficacy of MBIs on burnout for HC is not conclusive; different meta-analysis and systematic reviews show different results [45]. In ICU samples, previous MBIs reported them to be effective only in terms of emotional exhaustion [14].

Regarding acceptability, participants rated the usefulness and acceptability of the program as high, but they showed low levels of adherence in terms of daily practice, lower than expected, and lower than showed in similar programs. However, there is no previous information about adherence in ICU and homecare samples. This factor is essential, as research has shown how home practice predicts improvements in MBIs [46] and could explain the lack of significance in some variables of the present study.

The result of the qualitative analysis showed how the objectives of the program were expressed by the participants. The themes selected were the increase of well-being, emotion regulation, awareness, savoring, knowledge acquisition, and self-care and care for others. Participants reported improvements in these six levels, and it is important to note that the four constituents of WTCP appear in the participants’ narrative about improvements: awareness (mindfulness), savoring (sustained positive emotions), emotion regulation (recovery from negative emotions), and care for self and others (pro-social behavior and generosity). In terms of well-being, the main theme of the treatment, it was expected for participants to report benefits and knowledge acquisition, associated with the high number of theories that were explained in the program.

This study shows different limitations, such as the small sample or the lack of follow-up measures. Additionally, the results were not controlled for age or number of attended sessions. Another important limitation is that the study was not pre-registered. The main aim was to increase the knowledge that needs to be clarified before progressing to a full randomized study, given that it was the first time that the WTCP program was tested. Furthermore, there are few studies on the use of contemplative-based interventions in ICU and homecare unit professionals, so we decided to perform a feasibility pilot study. However, we could not randomize the sample due to the characteristics of the job in the ICU and homecare units. These limitations reduce the generalizability of the results, and there is a need to increase the effort to test the efficacy of this kind of intervention in this sample. Pilot studies are not recommended to test the efficacy of interventions, given that power analysis could be biased [47].

## 5. Conclusions

In conclusion, our results identified several potential benefits of the novel WTCP intervention, including a significant decrease in the frequency of negative emotions, a significant increase in self-compassion, and an increase in personal accomplishment in a group of healthcare professionals. Additionally, participants rated the usefulness and acceptability of the program as high. As regards the qualitative aspect of the study, participants reported improvements in well-being, emotion regulation, awareness, savoring, knowledge acquisition, and self-care and care for others. In all, and according to our results, WTCP can be a useful tool to address healthcare personnel burnout and wellbeing. The COVID-19 pandemic has posed a major risk to healthcare workers’ wellbeing as they have been particularly exposed due to being frontline care providers. Providing professionals with education and training to maintain an optimal level of wellbeing is key for healthcare systems and quality of care. Additionally, WTCP is a promising intervention in this context.

## Figures and Tables

**Table 1:** Descriptive statistics for the control and intervention groups.

Variable	Categories	Control Group (*n* = 15)		Intervention Group (*n* = 19)	
		M	SD	M	SD
Age		40.53	14.45	44.00	8.79
		**N**	**%**	**N**	**%**
Gender	Woman	14	93.3	16	84.2
	Man	1	6.7	2	10.5
	Missing	0	0.0	1	5.3
Profession	Physician	11	73.3	9	47.4
	Doctor	3	20.0	7	36.8
	Others (nursing assistants)	1	6.7	3	15.8
	Missing	0	0.0	0	0.0
Work unit	Intensive care	15	100.0	14	73.7
	Home care	0	0.0	4	21.1
	Missing	0	0.0	1	5.3

**Table 2:** *t*-tests for baseline group comparison in the main outcomes.

Instrument	Dimension	*t*	*df*	*p*
Difficulties in Emotion Regulation Scale (DERS)	Awareness	1.180	32	0.247
	Clarity	1.647	32	0.109
	Goals	1.389	32	0.174
	Impulse	1.411	32	0.168
	Non-acceptance	1.878	32	0.070
	Strategies	2.188	32	0.036
Modified Differential Emotions Scale (m-DES)	Positive emotions	3.569	32	0.001
	Negative emotions	1.744	32	0.091
Maslach Burnout Inventory (MBI)	Exhaustion	1.223	32	0.230
	Depersonalization	0.838	32	0.408
	Personal accomplishment	4.043	32	<0.001
Self-compassion Scale (SCS)	Positive self-compassion	1.057	32	0.298
	Negative self-compassion	0.700	32	0.489

Notes: *df* = degrees of freedom.

**Table 3:** Follow-up ANOVAs for the effects of time, group, and their interaction on the dependent variables.

Variables	Categories	Time					Group					Time * Group				
		*df_eff_*	*df_err_*	*F*	*p*	*η* ^2^	*df_eff_*	*df_err_*	*F*	*p*	*η* ^2^	*df_eff_*	*df_err_*	*F*	*p*	*η* ^2^
DERS	Awareness	1	32	0.007	0.936	0.000	1	32	2.543	0.121	0.074	1	32	0.477	0.495	0.015
	Clarity	1	32	0.682	0.415	0.021	1	32	5.289	0.028	0.142	1	32	0.682	0.415	0.021
	Goals	1	32	0.110	0.742	0.003	1	32	0.549	0.464	0.017	1	32	3.215	0.082	0.091
	Impulse	1	32	3.971	0.055	0.110	1	32	1.801	0.189	0.053	1	32	0.133	0.718	0.004
	Non-acceptance	1	32	2.953	0.095	0.084	1	32	2.971	0.094	0.085	1	32	0.312	0.580	0.010
	Strategies	1	32	0.303	0.586	0.009	1	32	5.292	0.028	0.142	1	32	0.020	0.889	0.001
m-DES	Positive emotions	1	31	9.977	0.004	0.243	1	31	7.557	0.010	0.196	1	31	2.146	0.153	0.065
	Negative emotions	1	31	54.665	<0.001	0.638	1	31	0.036	0.851	0.001	1	31	15.466	<0.001	0.333
MBI	Exhaustion	1	28	2.850	0.103	0.092	1	28	0.051	0.823	0.002	1	28	0.057	0.813	0.002
	Depersonalization	1	28	0.386	0.539	0.014	1	28	0.000	0.995	0.000	1	28	0.000	0.985	0.000
	Personal accomplishment	1	28	0.048	0.828	0.002	1	28	28.610	<0.001	0.505	1	28	4.483	0.043	0.138
SCS	Positive self-compassion	1	28	0.245	0.625	0.009	1	28	9.563	0.004	0.255	1	28	4.218	0.049	0.131
	Negative self-compassion	1	28	0.961	0.335	0.033	1	28	0.956	0.337	0.033	1	28	0.013	0.912	0.000

*Notes: df_eff_* = degrees of freedom of the effect; *df_err_* = degrees of freedom of the error.

**Table 4:** Variable means and standard deviations for the two time points (before and after the interventions) for the control and the intervention group.

Variable	Categories	Control Group				Intervention Group			
		Pre-Test		Post-Test		Pre-Test		Post-Test	
		M	SD	M	SD	M	SD	M	SD
DERS	Awareness	2.06	0.83	2.15	0.82	1.77	0.61	1.70	0.76
	Clarity	2.35	1.25	2.35	1.19	1.77	0.80	1.54	0.57
	Goals	2.84	1.33	2.64	1.43	2.32	0.84	2.61	0.99
	Impulse	2.13	1.08	2.40	1.27	1.68	0.77	2.07	0.72
	Non-acceptance	3.10	1.14	2.75	1.37	2.40	1.01	2.22	0.96
	Strategies	2.60	1.24	2.51	1.29	1.82	0.81	1.77	0.73
m-DES	Positive emotions	2.64	0.77	2.43	0.90	3.41	0.58	2.85	0.58
	Negative emotions	1.89	1.09	1.48	0.77	2.32	0.54	0.97	0.43
MBI	Exhaustion	9.00	5.33	6.35	4.44	10.68	4.32	6.21	3.73
	Depersonalization	5.00	2.25	3.71	2.43	5.84	3.00	3.26	2.28
	Personal accomplishment	15.21	2.25	13.07	4.02	18.20	2.14	15.89	1.76
SCS	Positive self-compassion	3.20	0.80	3.14	0.96	3.41	0.85	3.71	0.75
	Negative self-compassion	2.83	1.08	2.83	1.10	3.00	0.85	2.64	0.93

**Table 5:** Summary of qualitative feedback: themes and sub-themes.

Themes and Sub-Themes	Example Quotes
Theme 1: Emotion regulation (a) Improved ability to regulate difficult emotions. (b) Reduced boycott of positive emotions. (c) Learning to relativize, take perspective, and accept adverse situations.	“at times when maybe I’ve felt bad, like last Saturday, I did a meditation and... and then I felt better, it’s like what we were saying the other day about opening the window, opening the window and ventilating. It does bring me back to a state of calmness that... normally I’m usually quite calm and it brings me back to my state that I feel good (s8)”. “I notice it in the boycotts of my happiness, I don’t boycott myself as much as I did before doing the course (s4)”. “it has helped me to visualise conflicts in a different way (s10)”.
Theme 2: Awareness (a) Being more aware in general. (b) Greater emotional or social awareness.	“it has helped me to become more aware (s7)”. “I notice when I have an emotion that is hurting me (s1)”. “I have learned to be more aware of everything I do, and that benefits me both by eliminating the negative aspects and by anchoring myself in the positive ones (s14)”.
Theme 3: Well-being (a) Improved mood, better mood. (b) Increased low-activation positive emotions. (c) Mental stability and balance. (d) More spontaneity. (e) Improved sleep quality. (f) Increased concentration.	“being in a better mood (s1)”. “of course when you see life in a different way with more calm, your relationship with others changes, it is clear (s9)”. “I think that this way I have achieved a bit of mental stability (s2)”. “I have managed to be more spontaneous at some point (s3)”. “I’m sleeping a little better than before (s7)”. “I was very absent-minded, and... it’s helped me to concentrate (s7)”.
Theme 4: Savoring (a) Appreciating and being more aware of good things such as valuing the little things or own strengths. (b) Increased gratitude. (c) Learning to differentiate the good from the bad.	“I have always been aware of those little things, but today I have been more, and I think it is thanks to the course (s4)”. “it has been very good for me to bring out some strengths that I had, that I didn’t believe I could have (s13)”. “to be grateful for all the good things I have, which is a lot (s4)”. “you channel the bad news and you always get a good part out of it (s4).
Theme 5: Self-care and care for others (a) Greater self-compassion and good treatment of oneself. (b) Greater compassion, altruism, and kindness towards others. (c) Improvement in interpersonal relationships. (d) Improvement in the family climate.	“I think I have learned to be less hard on myself, to allow myself to have those defects that I didn’t allow myself to have before (s2)”. “at some point I have been more altruistic (s3)”. “above all by being more patient and tolerant with other people (s6)”. “where I have seen the most productivity from the course is in my relationship with people, this does not only include people at work, there is my partner, there is my mother, there is my sister, there is the world in general that you relate to and sure, sure there is (s10)”. “not only to stay in the intention, but also to do the action, especially with my family, because I have dedicated the course to my sister and my mother and I’m glad, because I already notice it (s6)”.
Theme 6: Knowledge acquisition (a) Acquisition of knowledge. (b) Increased self-knowledge.	“In the course we go very deeply into mindfulness, we practice with different techniques and so on, and then, apart from that, it has given me other knowledge (s3)”. “It has also helped me to get to know myself (s11)”.

## Data Availability

The data that support the findings of this study are available from the corresponding author upon reasonable request.

## Supplementary Materials

The following supporting information can be downloaded at: https://www.mdpi.com/article/10.3390/ijerph192013137/s1, Table S1: Summary of WTCP sessions (Cebolla & Alvear, 2019).
