# Supplementary material for: Wellbeing Training Based on Contemplative Practices in a Sample of Intensive Care and Homecare Professionals: A Pilot and Feasibility Non-Randomized Clinical Trial
Source: Int J Environ Res Public Health. 2022 Oct 12;19(20):13137. doi: 10.3390/ijerph192013137 (PMC9603612; doi:10.3390/ijerph192013137)
Supplement: Supplementary file 1 [file ijerph-19-13137-s001.zip › ijerph-1902717-supplementary.pdf]

SUPPLEMENTARY MATERIAL

Table S1. Summary of WTCP sessions (Cebolla & Alvear, 2019)

Session num.	Session name	Session content
1	Motivation and orientation to happiness	• Introduction to the course and the group<br>• Theoretical bases that support the training<br>• The importance of motivation<br>• Introduction imagination-based meditations
2	Mindfulness or the importance of attention	• Introduction to the first constituent: Mindfulness<br>• Attention regulation and meditation
3	Mindfulness or the importance of attention II	• The obstacles to its practice<br>• Inquiring the experiences
4	Empowering positive emotions	• Learning about positive emotions and their classification<br>• Acquisition and training of the ability to savor pleasant experiences from the past, present, and future<br>• Learning and practice of a positive emotion: Gratitude
5	Orienting the mind to the character strengths	• Learning about the character strengths and their classification in order to detect them more easily in daily life<br>• Identify one's five main strengths and the group's main strengths<br>• Learn to carry out behaviors and activities linked to personal strengths
6	Recovering from adversity: Regulating negative emotions	• Learning the role of negative emotions for psychological well-being<br>• Psychological skills' training to maintain well-being even in times of adversity<br>• Introduction to self-compassion<br>• Promotion and training kind treatment strategies through generative practices
7	From compassion to generosity: Happiness from surrender	• Knowing the importance of generosity, altruism, and compassion in achieving well-being<br>• To train the techniques for its development
8	Living the rest of your life with meaning	• Closing of the program and conclusions in order to continue the practice

Qualitative analysis

Theme 1: Emotion regulation.

In this first theme, the main element was the improved ability to regulate emotions reported by several participants. Understanding that sub-themes are essentially themes within a theme, three sub-themes were identified: (a) improved ability to regulate difficult emotions such as anger, anxiety, distress, or stress, (b) reduced boycott of positive emotions, and (c) learning to relativize, take perspective, and accept adverse situations.

Some of the participants reported that they had improved their ability to regulate difficult emotional states such as anger or anxiety. Thus, one participant pointed out that "at times when maybe I've felt bad, like last Saturday, I did a meditation and... and then I felt better, it's like what we were saying the other day about opening the window, opening the window and ventilating. It does bring me back to a state of calmness that... normally I'm usually quite calm and it brings me back to my state in which I feel good (s8)". Others found that the WTCP intervention had helped them decrease the boycotts directed at their own positive emotions, as one participant illustrated: "I notice it in the boycotts of my happiness, I don't boycott myself as much as I did before taking the course (s4)". There were also those who said that the training had helped them learn to relativize, to take a different perspective on adversities: "it has helped me to visualize conflicts in a different way (s10)".

Theme 2: Awareness.

Some of the participants commented that they had increased their ability to be aware. Within this theme, two sub-themes were detected: being more aware in general, and greater emotional or social awareness. Regarding being more aware in general, general statements such as "it has helped me to become more aware (s7)", or "it has helped me to be more aware of things" were

collected. Others indicated increased awareness at a more specific level, the following is an example of increased emotional awareness: "I notice when I have an emotion that is hurting me (s1)". It is remarkable how some subjects related increased awareness to the resulting benefit, "I have learned to be more aware of everything I do, and that benefits me both by eliminating the negative aspects and by anchoring myself in the positive ones (s14)".

Theme 3: Well-being.

Increased psychological well-being as a benefit of the training was overall reported among the participants. However, the perspective, the experiential experience, and the level of concreteness when talking about increased well-being was different among them. From this plurality of experiences we detected six sub-themes: (a) improved mood, better mood, (b) increased low-activation positive emotions such as calmness, tranquility, and peace, (c) mental stability and balance, (d) more spontaneity, (e) improved sleep quality, and (d) increased concentration.

There were participants who reported "being in a better mood (s1)", while others revealed that they perceived an increase in positive emotions of low activation such as tranquility or calmness "of course when you see life in a different way with more calm, your relationship with others changes, it is clear (s9)". There were also those who perceived greater mental stability, "I think that this way I have achieved a bit of mental stability (s2)" and greater spontaneity, "I have managed to be more spontaneous at some point (s3)".

In other participants the increase in well-being was related to improved sleep quality, "I'm sleeping a little better than before (s7)" and with increased concentration, "I was very absent-minded, and... it's helped me to concentrate (s7)".

Theme 4: Savoring.

Another type of benefit derived from the WTCP intervention that participants indicated was an increased ability to value the good. Three differential sub-themes arose: a) appreciating and being more aware of good things such as valuing the little things or one's strengths, b) increased gratitude, and c) learning to differentiate the good from the bad.

Appreciating and being more aware of the little good things that happen in everyday life was a repeated message, with one participant noting that "I have always been aware of those little things, but today I have been more, and I think it is thanks to the course (s4)". Other users talked about the benefit of detecting and valuing the strengths already existing in themselves, "it has been very good for me to bring out some strengths that I had, that I didn't believe I could have (s13)".

Appreciation of what is good can also come from other perspectives, and gratitude was one way of doing this, with one participant saying that she had learned "to be grateful for all the good things I have, which is a lot (s4)". It was also mentioned that they had learned to revalue adverse experiences by detecting something good in the bad, "you channel the bad news, and you always get a good part out of it (s4)".

Theme 5: Self-care and care for others.

Something that was widely noted by the participants was the improvement in interpersonal relationships and in the relationship with oneself. We refer to this theme as self-care and care for others, although we established four conceptually distinct sub-themes: a) greater self-compassion and good treatment of oneself, b) greater compassion, altruism and kindness towards others, c) improvement in interpersonal relationships, and d) improvement in the family climate.

On the one hand, some participants highlighted that they had noticed an increase in self-care and self-compassion, "I think I have learned to be less hard on myself, to allow myself to have those defects that I didn't allow myself to have before (s2)". On the other hand, they also learned to be more compassionate and altruistic towards others, one participant said that "at some point I have been more altruistic (s3)".

Another widely shared aspect was the improvement in interpersonal relationships, both at work and in friendships. One person commented that she had noticed the benefit of the program "above all by being more patient and tolerant with other people (s6)". It seems that the program also had an impact on participants' families, some reported an improvement in both work relationships and family climate: "where I have seen the most productivity from the course is in my relationship with people, this does not only include people at work, there is my partner, there is my mother, there is my sister, there is the world in general that you relate to and sure, sure there is (s10)". Another participant shared that the intervention had helped them: "not only to stay in the intention, but also to do the action, especially with my family, because I have dedicated the course to my sister and my mother and I'm glad, because I already notice it (s6)", in this case referring to a specific practice of ego-centeredness trained in the intervention.

Theme 6: Knowledge acquisition.

As in most intervention groups, in the WTCP intervention there was also a structured teaching-learning process in which participants reported having acquired certain knowledge. In this case, according to the data collected, we configured the acquisition of knowledge into two sub-themes: the acquisition of knowledge and reflection on it, and increased self-knowledge.

We found interviewees who referred to the acquisition of new knowledge, both by going in depth into a subject they already knew and by acquiring new psychological skills, "in the course we go very deep into mindfulness, we practice with different techniques and so on, and then,

apart from that, it has given me other knowledge (s3)". Finally, other people related this acquisition of knowledge to greater self-knowledge, "it has also helped me to get to know myself (s11)".
